# Sequential Covariance Intersection Fusion Robust Time-Varying Kalman Filters with Uncertainties of Noise Variances for Advanced Manufacturing

**DOI:** 10.3390/mi13081216

**Published:** 2022-07-29

**Authors:** Wenjuan Qi, Shigang Wang

**Affiliations:** School of Mechanical and Electrical Engineering, Heilongjiang University, Harbin 150080, China; 2015072@hlju.edu.cn

**Keywords:** multisensor data fusion, sequential covariance intersection fusion, robust Kalman filter, robust accuracy, uncertain noise variance, convergence

## Abstract

This paper addresses the robust Kalman filtering problem for multisensor time-varying systems with uncertainties of noise variances. Using the minimax robust estimation principle, based on the worst-case conservative system with the conservative upper bounds of noise variances, the robust local time-varying Kalman filters are presented. Further, the batch covariance intersection (BCI) fusion and a fast sequential covariance intersection (SCI) fusion robust time-varying Kalman filters are presented. They have the robustness that the actual filtering error variances or their traces are guaranteed to have a minimal upper bound for all admissible uncertainties of noise variances. Their robustness is proved based on the proposed Lyapunov equations approach. The concepts of the robust and actual accuracies are presented, and the robust accuracy relations are proved. It is also proved that the robust accuracies of the BCI and SCI fusers are higher than that of each local Kalman filter, the robust accuracy of the BCI fuser is higher than that of the SCI fuser, and the actual accuracies of each robust Kalman filter are higher than its robust accuracy for all admissible uncertainties of noise variances. The corresponding steady-state robust local and fused Kalman filters are also presented for multisensor time-invariant systems, and the convergence in a realization between the local and fused time-varying and steady-state Kalman filters is proved by the dynamic error system analysis (DESA) method and dynamic variance error system analysis (DVESA) method. A simulation example is given to verify the robustness and the correctness of the robust accuracy relations.

## 1. Introduction

The multisensor information fusion Kalman filtering has wide applications in many high-technology fields, such as advanced manufacturing systems, mechanical industrial robots, unmanned aircraft vehicles, tracking, signal processing, remaining useful life prediction of rolling element bearings [1,2,3], improved tracking and docking of industrial mobile robots [4,5,6,7], and so on. Rolling bearings are the key components of rotating machinery, thus, the prediction of remaining useful life (RUL) is vital in condition-based maintenance (CBM). Reference 1 proposes a new method for RUL predictions of bearings based on time-varying Kalman filter, which can automatically match different degradation stages of bearings and effectively realize the prediction of RUL. Industrial mobile robots are widely used in advanced manufacturing technology systems; ref. [2] used the unscented Kalman filter to improved tracking and docking of industrial mobile robots vision-based kinematics calibration.

The basic assumption for classical Kalman filtering is that the model parameters and noise variances are exactly known, but in many practical applications, such assumption doesn’t always hold. In the presence of these uncertainties, the Kalman filters may not be robust against uncertainties, or may be divergent [8], or the performance of the filters is degraded. In order to solve the filtering problems for uncertain systems, in recent years several results have been derived on the design of various robust Kalman filters. The so-called robust Kalman filtering problem is to find a Kalman filter whose actual filtering error variances, or their traces, are guaranteed to have a minimal or less-conservative upper bound for all admissible uncertainties. There are basically two approaches to solve this problem for the systems with uncertainties of model parameters: one is the Riccati equation approach [8,9,10,11]; the other is the linear matrix inequality (LMI) approach [8,12,13].

The uncertain systems exist widely in control engineering and signal processing. So far, these robust Kalman approaches are only suitable to the systems with the uncertainty of model parameters, while the uncertainties of noise variances are seldom considered. Many results are limited to design the robust Kalman filters for single sensor systems, while the multisensor fusion robust Kalman filters are seldom proposed [14,15,16,17], and the robustness analysis problem was not solved.

The multisensor data fusion problem is to find a fused state estimator based on the local sensor measurement information or the local state estimators such that its accuracy is higher than that of each local state estimator [18,19,20]. There exist two kinds of fusion methods, which are called the centralized and distributed fusion methods depending on whether raw data are used directly for fusion or not [19]. For the centralized fusion method, all the measurement data from local sensors are carried to the fusion center which can give the global optimal fusion estimate, but its disadvantage is to require a large computation burden. The distributed fusion method can give the globally optimal or suboptimal state estimation by combing the local state estimators [20,21,22], whose advantages are that it can reduce the computation burden and can realize fault detection and isolation more conveniently. Under the unbiased linear minimum variance rule (ULMV), there are three distributed optimal fusion rules weighted by matrices, diagonal matrices, and scalars, respectively, which were presented in [20,23].

It is well known that to compute the optimal weights requires knowing the cross-covariance among the local Kalman filtering errors [20,21,22,23]; however, in many practical applications, the variances and cross-covariances of the local filtering errors are unknown or uncertain, or the computation of the cross-covariances is very complex and difficult [21,24]. In order to overcome the above limitation, the covariance intersection (CI) fusion method has been presented in [25,26,27,28,29] and has been widely applied in many fields; for example, the simultaneous localization and mapping (SLAM) [29], remote sensing [30], rocket tracking [31], spacecraft estimation [32], vehicle localization [33] and so on. The CI fuser is obtained by the convex combination of the local estimators, and it has the advantages that the fused estimation problems can be solved for multisensor systems with unknown variances and cross-covariances of local filtering errors, and the computation of the cross-covariances is completely avoided. However, its disadvantage is that the conservative upper bounds of the unknown local filtering error variances are assumed to be known, i.e., the consistent estimation problem of the unknown local filtering error variances was not solved.

Based on the batch processing method, the batch covariance intersection (BCI) fusion Kalman filter with exactly known model parameters and noise variances is presented [22]; this needs to solve the high-dimensional nonlinear optimization problem, so that a larger computation burden and higher complexity are required. In order to reduce the computation burden and complexity by the sequential procession method, a sequential covariance intersection (SCI) fusion Kalman filter is presented in [34] for multisensor systems with noise variances to be known exactly.

In this paper, we will focus on the covariance intersection (CI) fused robust Kalman filtering for multisensor systems with uncertainties of noise variances. A robust CI fusion Kalman filtering theory and methodology are presented. Compared with references [22,25,26,27,28,29,34], the main contributions are as follows:

1. In Section 2 and Section 3, a new methodology for designing the robust local and CI fused Kalman filters is presented for multisensor time-varying systems with uncertain noise variances, according to the minimax robust estimation rule [35,36]. Its basic principle is that based on the worst-case conservative system with the conservative upper bound of noise variances, applying the ULMV optimal estimation rule, the conservative local and CI fused Kalman filters with unavailable conservative measurements are obtained, and then replacing the conservative measurements with the actual measurements yields the robust local and CI fused Kalman filters. The classical optimal Kalman filtering methodology [22,34] is developed. The disadvantage of the original CI fusion methodology [25,26,27,28,29] is overcome where the conservative upper bounds of the local filtering error variances are assumed to be known. Hence the robust local Kalman filters are presented, which provide the conservative upper bounds of the local filtering error variances;
2. In Section 3, the robust time-varying BCI and SCI fused Kalman filters with uncertain noise variances are presented. The steady-state optimal local, BCI and SCI fused Kalman filters [22,34] with exactly known noise variances are developed;
3. In the process of proving Theorems 1 and 3, a Lyapunov equation method for the robustness analysis is presented by which the robustness of the local and CI fused Kalman filters is proved. Its basic principle is that the problem of proving the robustness is converted into that of deciding the positive-definiteness of the solution of a Lyapunov equation;
4. In Section 4, the concept of robust accuracy with respect to uncertainties of noise variances is presented, and the robust accuracy relations among the local, BCI and SCI fused Kalman filters with exactly known noised variances [22,34] are extended. The concept of robustness with respect to uncertain noise variances is presented, and the concept of consistency [25,26] is extended;
5. In Section 5, for the multisensor time-invariant system with uncertain noise variances, the robust steady-state local, BCI and SCI fusion Kalman filters are also presented by replacing time-varying gains, variances and cross-covariances with their limits, respectively;
6. Using lemma 1–3, in Theorem 7, the convergence in a realization of the local and fused time-varying and steady-state robust Kalman filters is proved by the dynamic error system analysis (DESA) method and the dynamic variance error system analysis (DVESA) method. To the best of our knowledge, it is presented for the first time;
7. In Section 7, simulation 1 gives the geometric interpretation of the robust accuracy relations based on the variance ellipses and a Monte Carlo simulation example shows the correctness of the proposed robust accuracy relations and gives the sensitivity analysis of the robust SCI fuser.

The remainder of this paper is organized as follows: In Section 2, we derive the local robust time-varying Kalman filter and prove its robustness. Section 3 gives the BCI and SCI fusion robust time-varying Kalman filters and the proof of their robustness. The accuracy analysis of the local and fused Kalman filters is presented in Section 4. Section 5 gives the robust local and fused steady-state Kalman filters and their convergence. The sensitivity problem is given in Section 6. Section 7 gives a Monte Carlo simulation example. The conclusions are given in Section 8. The frequently used notations in the paper are shown in Table 1.

## 2. Local Robust Time-Varying Kalman Filters

Consider the following multisensor uncertain time-varying system with uncertainties of noise variances

(1) x(t+1)=Φ(t)x(t)+Γ(t)w(t)

(2) $$y_{i}\left(t\right)=H_{i}\left(t\right)x\left(t\right)+v_{i}\left(t\right),\;i=1,\cdots ,L$$

where $x\left(t\right)\in R^{n}$ is the state, L is the number of sensors, $y_{i}\left(t\right)\in R^{m_{i}}$ is the measurement of the ith subsystem, $w\left(t\right)\in R^{r}$ is the input noise and $v_{i}\left(t\right)\in R^{m_{i}}$ is the measurement noise of the ith sensor. Φ(t), Γ(t) and $H_{i}\left(t\right)$ are known time-varying matrices with appropriate dimensions.

**Assumption** **1.** w(t)*and*$v_{i}\left(t\right)$*are uncorrelated white noises with zeros mean and unknown uncertain true variances*$\overset{¯}{Q}\left(t\right)$*and*${\overset{¯}{R}}_{i}\left(t\right)$, *respectively*.

(3) $$E[\left(\begin{array}{c} w\left(t\right)\\ v_{i}\left(t\right) \end{array}\right){\left(\begin{array}{cc} w\left(k\right) & v_{j}\left(k\right) \end{array}\right)}^{Τ}]=\left[\begin{array}{cc} \overset{¯}{Q}\left(t\right) & 0\\ 0 & {\overset{¯}{R}}_{i}\left(t\right)\delta_{ij} \end{array}\right]\delta_{tk}\;$$

**Assumption 2.** Q(t)*and*$R_{i}\left(t\right)$*are known conservative upper bounds of*$\overset{¯}{Q}\left(t\right)$*and*${\overset{¯}{R}}_{i}\left(t\right)$, *respectively, i.e.,*

(4) $$\overset{¯}{Q}\left(t\right)\leq Q\left(t\right),\;{\overset{¯}{R}}_{i}\left(t\right)\leq R_{i}\left(t\right),\;\forall t,i=1,\cdots ,L\;$$

**Assumption** **3.** *The initial state*x(0)*is independent of*w(t)*and*$v_{i}\left(t\right)$, *and has mean value*μ*and unknown uncertain true variance*$\overset{¯}{P}\left(0|0\right)$*which satisfies*

(5) $$\overset{¯}{P}\left(0|0\right)\leq P\left(0|0\right)\;$$

*where* P(0|0) *is a known conservative upper bound of* $\overset{¯}{P}\left(0|0\right)$.

Based on the ith sensor, for the worst-case conservative multisensor system (1) and (2) with the known conservative upper bounds Q(t) and $R_{i}\left(t\right)$ of noise variances, the conservative local optimal time-varying Kalman filters are given by [20]

(6) $${\hat{x}}_{i}\left(t|t\right)=\Psi_{i}\left(t\right){\hat{x}}_{i}\left(t-1|t-1\right)+K_{i}\left(t\right)y_{i}\left(t\right),\;i=1,\cdots ,L$$

(7) $$\Psi_{i}\left(t\right)=\left[I_{n}-K_{i}\left(t\right)H_{i}\left(t\right)\right]\Phi \left(t\right)$$

(8) $$K_{i}\left(t\right)=P_{i}\left(t|t-1\right)H_{i}^{T}\left(t\right)Q_{\varepsilon i}^{-1}\left(t\right)$$

(9) $$Q_{\varepsilon i}\left(t\right)=H_{i}\left(t\right)P_{i}\left(t|t-1\right)H_{i}^{T}\left(t\right)+R_{i}\left(t\right)$$

(10) $$P_{i}\left(t|t-1\right)=\Phi \left(t-1\right)P_{i}\left(t-1|t-1\right)\Phi_{}^{T}\left(t-1\right)+\Gamma \left(t-1\right)Q\left(t-1\right)\Gamma^{T}\left(t-1\right)$$

(11) $$P_{i}\left(t|t\right)=\left[I_{n}-K_{i}\left(t\right)H_{i}\left(t\right)\right]P_{i}\left(t|t-1\right)$$

(12) $$\begin{array}{l} P_{ij}\left(t|t\right)=\Psi_{i}\left(t\right)P_{ij}\left(t-1|t-1\right)\Psi_{j}^{T}\left(t\right)+\left[{Ι}_{n}-{Κ}_{i}\left(t\right)H_{i}\left(t\right)\right]\\ \times \Gamma \left(t-1\right)Q\left(t-1\right)\Gamma^{T}\left(t-1\right){\left[I_{n}-K_{j}\left(t\right)H_{j}\left(t\right)\right]}^{T}+K_{i}\left(t\right)R_{ij}\left(t\right)K_{j}^{T}\left(t\right)\delta_{ij} \end{array}$$

(13) $${\overset{¯}{P}}_{i}\left(t|t\right)=E\left[{\tilde{x}}_{i}\left(t|t\right){\tilde{x}}_{i}^{Τ}\left(t|t\right)\right],\;{\overset{¯}{P}}_{ij}\left(t|t\right)=E\left[{\tilde{x}}_{i}\left(t|t\right){\tilde{x}}_{j}^{Τ}\left(t|t\right)\right]$$

(14) $${\tilde{x}}_{i}\left(t|t\right)=x\left(t\right)-{\hat{x}}_{i}\left(t|t\right)$$

From (1) and (6), the actual filtering errors are

(15) $${\tilde{x}}_{i}\left(t|t\right)=\Psi_{i}\left(t\right)\tilde{x}\left(t-1|t-1\right)+\left(I_{n}-K_{i}\left(t\right)H_{i}\left(t\right)\right)\Gamma \left(t\right)w\left(t-1\right)-K_{i}\left(t\right)v_{i}\left(t\right)$$

From (15), according to Assumptions 1–3, and noting that w(t) and $v_{i}\left(t\right)$ are uncorrelated with ${\tilde{x}}_{i}\left(t|t\right)$, the actual filtering error variance and cross-covariances are given by the Lyapunov equations

(16) $$\begin{array}{l} {\overset{¯}{P}}_{ij}\left(t|t\right)=\Psi_{i}\left(t\right){\overset{¯}{P}}_{ij}\left(t-1|t-1\right)\Psi_{j}^{T}\left(t\right)+\left[{Ι}_{n}-{Κ}_{i}\left(t\right)H_{i}\left(t\right)\right]\\ \times \Gamma \left(t-1\right)\overset{¯}{Q}\left(t-1\right)\Gamma^{T}\left(t-1\right){\left[I_{n}-K_{j}\left(t\right)H_{j}\left(t\right)\right]}^{T}+K_{i}\left(t\right){\overset{¯}{R}}_{ij}\left(t\right)K_{j}^{T}\left(t\right)\delta_{ij} \end{array}$$

with the initial values ${\overset{¯}{P}}_{ij}\left(0|0\right)=\overset{¯}{P}\left(0|0\right)$ and ${\overset{¯}{P}}_{ii}\left(t|t\right)={\overset{¯}{P}}_{i}\left(t|t\right)$.

**Theorem** **1.** *For multisensor uncertain system (1) and (2) with Assumptions 1–3, the actual local Kalman filters (6) is robust in the sense that for all admissible variances*$\overset{¯}{Q}\left(t\right)$*and*${\overset{¯}{R}}_{i}\left(t\right)$*satisfying (4) and*$\overset{¯}{P}\left(0|0\right)\leq P\left(0|0\right)$*for arbitrary time*t, we have

(17) $${\overset{¯}{P}}_{i}\left(t|t\right)\leq P_{i}\left(t|t\right),\;i=1,\cdots ,L\;$$

*and* $P_{i}\left(t|t\right)$ *are the minimal upper bounds of* ${\overset{¯}{P}}_{i}\left(t|t\right)$. *Hence, they are called the robust local Kalman filters.*

**Proof.**  Defining $\Delta P_{i}\left(t|t\right)=P_{i}\left(t|t\right)-{\overset{¯}{P}}_{i}\left(t|t\right)$, subtracting (16) from (12) yields the Lyapunov equations

(18) $$\Delta P_{i}\left(t|t\right)=\Psi_{i}\left(t\right)\Delta P_{i}\left(t-1|t-1\right)\Psi_{i}^{T}\left(t\right)+U_{i}\left(t\right)$$

(19) $$\begin{array}{l} U_{i}\left(t\right)=\left[{Ι}_{n}-{Κ}_{i}\left(t\right)H_{i}\left(t\right)\right]\Gamma \left(t-1\right)\left(Q\left(t-1\right)-\overset{¯}{Q}\left(t-1\right)\right)\Gamma^{T}\left(t-1\right){\left[I_{n}-K_{i}\left(t\right)H_{i}\left(t\right)\right]}^{T}\\ +K_{i}\left(t\right)\left(R_{i}\left(t\right)-{\overset{¯}{R}}_{i}\left(t\right)\right)K_{i}^{T}\left(t\right) \end{array}$$

Applying (4) yields that $U_{i}\left(t\right)\geq 0$. From (5), we have $\Delta P_{i}\left(0|0\right)=P\left(0|0\right)-\overset{¯}{P}\left(0|0\right)\geq 0$. Hence from (18), we have $\Delta P_{i}\left(1|1\right)\geq 0$. Applying the mathematical induction method yields $\Delta P_{i}\left(t|t\right)\geq 0$, for all time t, i.e., the inequalities (17) hold. If $P_{i}^{*}\left(t|t\right)$ is another upper bound, then for all admissible $\overset{¯}{Q}\left(t\right)\leq Q\left(t\right)$ and ${\overset{¯}{R}}_{i}\left(t\right)\leq R_{i}\left(t\right)$, we have ${\overset{¯}{P}}_{i}\left(t|t\right)\leq P_{i}^{*}\left(t|t\right)$. Taking $\overset{¯}{Q}\left(t\right)=Q\left(t\right),$ ${\overset{¯}{R}}_{i}\left(t\right)=R_{i}\left(t\right)$, from (12) and (16), we have $P_{i}\left(t|t\right)={\overset{¯}{P}}_{i}\left(t|t\right)\leq P_{i}^{*}\left(t|t\right)$. This means that $P_{i}\left(t|t\right)$ is the minimal upper bounds of ${\overset{¯}{P}}_{i}\left(t|t\right)$. The proof is completed. □

**Remark** **1.** *The robustness (17) is different from the consistency or non-divergent estimation [23]. The robustness means that the inequality (17) holds for all admissible uncertain* $\overset{¯}{Q}\left(t\right)$ *and* ${\overset{¯}{R}}_{i}\left(t\right)$ *satisfying (4), while the consistency means that for a fixed* $\overset{¯}{Q}\left(t\right)$ *and* ${\overset{¯}{R}}_{i}\left(t\right)$ *, the inequality (17) holds.*

## 3. The CI Fusion Robust Time-Varying Kalman Filter

### 3.1. The BCI Fusion Robust Time-Varying Kalman Filter

For the two-sensor uncertain systems with the Assumptions 1–3, applying the CI fused algorithm [20,21,22,23], the actual CI fusion time-varying Kalman filter with the conservative upper bounds Q(t) and $R_{i}\left(t\right)$ of noise variances is presented as following

(20) $${\hat{x}}_{CI}\left(t|t\right)=P_{CI}\left(t|t\right)\left[\omega \left(t\right)P_{1}^{-1}\left(t|t\right){\hat{x}}_{1}\left(t|t\right)+\left(1-\omega \left(t\right)\right)P_{2}^{-1}\left(t|t\right){\hat{x}}_{2}\left(t|t\right)\right]$$

(21) $$P_{CI}\left(t|t\right)={\left[\omega \left(t\right)P_{1}^{-1}\left(t|t\right)+\left(1-\omega \left(t\right)\right)P_{2}^{-1}\left(t|t\right)\right]}^{-1},\;\omega \left(t\right)\in \left[0,1\right]$$

where ${\hat{x}}_{i}\left(t|t\right)$ are the robust local Kalman filters given in Theorem 1. The weight ω(t) minimizes the cost function J as

(22) $$\underset{}{\min}J=\underset{\omega \left(t\right)\in \left[0,1\right]}{\min}\mathrm{tr}P_{CI}\left(t|t\right)=\underset{\omega \left(t\right)\in \left[0,1\right]}{\min}\mathrm{tr}\left\{{\left[\omega \left(t\right)P_{1}^{-1}\left(t|t\right)+\left(1-\omega \left(t\right)\right)P_{2}^{-1}\left(t|t\right)\right]}^{-1}\right\}$$

When the number of the sensors is larger than two, i.e., L≥3. The actual batch covariance intersection (BCI) fusion Kalman filter is presented by the convex combination [26,35] as

(23) $${\hat{x}}_{BCI}\left(t|t\right)=P_{BCI}\left(t|t\right)\sum_{i=1}^{L}\omega_{i}\left(t\right)P_{i}^{-1}\left(t|t\right){\hat{x}}_{i}\left(t|t\right)$$

(24) $$P_{BCI}\left(t|t\right)={\left[\sum_{i=1}^{L}\omega_{i}\left(t\right)P_{i}^{-1}\left(t|t\right)\right]}^{-1},\;\sum_{i=1}^{L}\omega_{i}\left(t\right)=1,0\leq \omega_{i}\left(t\right)\leq 1$$

where ${\hat{x}}_{i}\left(t|t\right)$ are the robust local Kalman filters, the weights $\omega_{i}\left(t\right)$ are determined by minimizing the performance index $J=\mathrm{tr}P_{BCI}\left(t|t\right)$ as

(25) $$\min J=\underset{\omega_{i}\left(t\right)}{\min}\mathrm{tr}P_{BCI}\left(t|t\right)=\underset{\begin{array}{l} \omega_{i}\left(t\right)\in \left[0,1\right]\\ \omega_{1}\left(t\right)+\cdots +\omega_{L}\left(t\right)=1 \end{array}}{\min}\mathrm{tr}\left\{{\left[\sum_{i=1}^{L}\omega_{i}\left(t\right)P_{i}^{-1}\left(t|t\right)\right]}^{-1}\right\}$$

which can be obtained by “fimincon” function in Matlab. This needs to solve a L-dimensional nonlinear convex optimization problem, so that the larger computation burden and higher complexity are required.

**Theorem** **2.** *The actual BCI fusion filtering error variance is given by*

(26) $${\overset{¯}{P}}_{BCI}\left(t|t\right)=P_{BCI}\left(t|t\right)\left[\sum_{i=1}^{L}\sum_{j=1}^{L}\omega_{i}\left(t\right)P_{i}^{-1}\left(t|t\right){\overset{¯}{P}}_{ij}\left(t|t\right)P_{j}^{-1}\left(t|t\right)\omega_{j}\left(t\right)\right]P_{BCI}\left(t|t\right)\;$$

*where* ${\overset{¯}{P}}_{ij}\left(t|t\right)$ *are computed by (16)*.

**Proof.**  From (24), we have

(27) $$x\left(t\right)=P_{BCI}\left(t|t\right)\left[\sum_{i=1}^{L}\omega_{i}\left(t\right)P_{i}^{-1}\left(t|t\right)\right]x\left(t\right)$$

Subtracting (27) from (23), we easily obtain the actual BCI fused filtering error

(28) $${\tilde{x}}_{BCI}\left(t|t\right)=P_{BCI}\left(t|t\right)\sum_{i=1}^{L}\omega_{i}\left(t\right)P_{i}^{-1}\left(t|t\right){\tilde{x}}_{i}\left(t|t\right)$$

which yields the formula (26). The proof is completed. □

**Theorem** **3.** *For multisensor uncertain system (1) and (2) with Assumptions 1–3, the actual BCI fusion time-varying Kalman filter (23)–(25) is robust in the sense that for all admissible uncertainties of noise variances*$\overset{¯}{Q}\left(t\right)$*and*${\overset{¯}{R}}_{i}\left(t\right)$*satisfying (4), we have*

(29) $${\overset{¯}{P}}_{BCI}\left(t|t\right)\leq P_{BCI}\left(t|t\right)\;$$

*and* $\mathrm{tr}P_{BCI}\left(t|t\right)$ *is the minimal upper bound of* $\mathrm{tr}{\overset{¯}{P}}_{BCI}\left(t|t\right)$. *We call (23)as the robust BCI fusion Kalman filter.*

**Proof.**  In order to prove (29), we only need to prove

(30) $$P_{BCI}\left(t|t\right)-{\overset{¯}{P}}_{BCI}\left(t|t\right)\geq 0$$

Pre-multiplying and post-multiplying (30) by $P_{BCI}^{-1}$, respectively, we have

(31) $$P_{BCI}^{-1}\left(t|t\right)-P_{BCI}^{-1}\left(t|t\right){\overset{¯}{P}}_{BCI}\left(t|t\right)P_{BCI}^{-1}\left(t|t\right)\geq 0$$

Substituting (24) and (26) into (31), we only need to prove

(32) $$\sum_{i=1}^{L}\omega_{i}\left(t\right)P_{i}^{-1}\left(t|t\right)-\sum_{i=1}^{L}\sum_{j=1}^{L}\omega_{i}\left(t\right)P_{i}^{-1}\left(t|t\right){\overset{¯}{P}}_{ij}\left(t|t\right)P_{j}^{-1}\left(t|t\right)\omega_{j}\left(t\right)\geq 0$$

From (17) for all admissible $\overset{¯}{Q}\left(t\right)$ and ${\overset{¯}{R}}_{i}\left(t\right)$ satisfying (4), we have

(33) $$P_{i}\left(t|t\right)-{\overset{¯}{P}}_{i}\left(t|t\right)\geq 0$$

Pre-multiplying and post-multiplying (33) by $P_{i}^{-1}$, respectively, we have

(34) $$P_{i}^{-1}\left(t|t\right)-P_{i}^{-1}\left(t|t\right){\overset{¯}{P}}_{i}\left(t|t\right)P_{i}^{-1}\left(t|t\right)\geq 0$$

From (32) and (34), we only need to prove

(35) $$\sum_{i=1}^{L}\omega_{i}\left(t\right)P_{i}^{-1}\left(t|t\right){\overset{¯}{P}}_{i}\left(t|t\right)P_{i}^{-1}\left(t|t\right)-\sum_{i=1}^{L}\sum_{j=1}^{L}\omega_{i}\left(t\right)P_{i}^{-1}\left(t|t\right){\overset{¯}{P}}_{ij}\left(t|t\right)P_{j}^{-1}\left(t|t\right)\omega_{j}\left(t\right)\geq 0$$

Applying the constraint $\sum_{i=1}^{L}\omega_{i}\left(t\right)=1$ yields that

(36) $$\sum_{i=1}^{L}\omega_{i}\left(t\right)P_{i}^{-1}\left(t|t\right){\overset{¯}{P}}_{i}\left(t|t\right)P_{i}^{-1}\left(t|t\right)=\sum_{i=1}^{L}\sum_{j=1}^{L}\omega_{i}\left(t\right)\omega_{j}\left(t\right)P_{i}^{-1}\left(t|t\right){\overset{¯}{P}}_{i}\left(t|t\right)P_{i}^{-1}\left(t|t\right)$$

Hence, we only need to prove

(37) $$\Delta =\sum_{i=1}^{L}\sum_{j=1}^{L}\omega_{i}\left(t\right)\omega_{j}\left(t\right)\left(P_{i}^{-1}\left(t|t\right){\overset{¯}{P}}_{i}\left(t|t\right)P_{i}^{-1}\left(t|t\right)-P_{i}^{-1}\left(t|t\right){\overset{¯}{P}}_{ij}\left(t|t\right)P_{j}^{-1}\left(t|t\right)\right)\geq 0$$

Exchanging the subscript symbol i with j in (37) yields

(38) $$\Delta =\sum_{j=1}^{L}\sum_{i=1}^{L}\omega_{j}\left(t\right)\omega_{i}\left(t\right)\left(P_{j}^{-1}\left(t|t\right){\overset{¯}{P}}_{j}\left(t|t\right)P_{j}^{-1}\left(t|t\right)-P_{j}^{-1}\left(t|t\right){\overset{¯}{P}}_{ji}\left(t|t\right)P_{i}^{-1}\left(t|t\right)\right)\geq 0$$

Adding (37) to (38) yields

(39) $$\begin{array}{l} 2\Delta =\sum_{i=1}^{L}\sum_{j=1}^{L}\omega_{i}\left(t\right)\omega_{j}\left(t\right)[P_{i}^{-1}\left(t|t\right){\overset{¯}{P}}_{i}\left(t|t\right)P_{i}^{-1}\left(t|t\right)+P_{j}^{-1}\left(t|t\right){\overset{¯}{P}}_{j}\left(t|t\right)P_{j}^{-1}\left(t|t\right)\\ \begin{array}{cc} & -P_{i}^{-1}\left(t|t\right){\overset{¯}{P}}_{ij}\left(t|t\right)P_{j}^{-1}\left(t|t\right)-P_{j}^{-1}\left(t|t\right){\overset{¯}{P}}_{ji}\left(t|t\right)P_{i}^{-1}\left(t|t\right)] \end{array}\\ \begin{array}{cc} & = \end{array}\sum_{i=1}^{L}\sum_{j=1}^{L}\omega_{i}\left(t\right)\omega_{j}\left(t\right)E[\left(P_{i}^{-1}\left(t|t\right){\tilde{x}}_{i}\left(t|t\right)-P_{j}^{-1}\left(t|t\right){\tilde{x}}_{j}\left(t|t\right)\right)\\ \begin{array}{cc} & \times {\left(P_{i}^{-1}\left(t|t\right){\tilde{x}}_{i}\left(t|t\right)-P_{j}^{-1}\left(t|t\right){\tilde{x}}_{j}\left(t|t\right)\right)}^{T}]\geq 0 \end{array} \end{array}$$

which yields Δ≥0, i.e., (29) holds. Taking the trace operation for (29) yields $\mathrm{tr}{\overset{¯}{P}}_{BCI}\left(t|t\right)\leq \mathrm{tr}P_{BCI}\left(t|t\right)$. Applying (25) yields that $\mathrm{tr}P_{BCI}\left(t|t\right)$ is minimal for all admissible $P_{BCI}\left(t|t\right)$ given in (24). The proof is completed. □

**Remark** **2.** *The proof of Theorem 3 is completely different from the proof in reference [20], where the noise variances are assumed to be exactly known, and the consistency is proved by the mathematical induction. The proof is also different from that in reference [36], where the consistency of the BCI fuser was only proved with the assumption that the local estimates are consistent, while the robustness problem was not proved.*

### 3.2. The SCI Fusion Robust Time-Varying Kalman Filter

**Figure 1:**
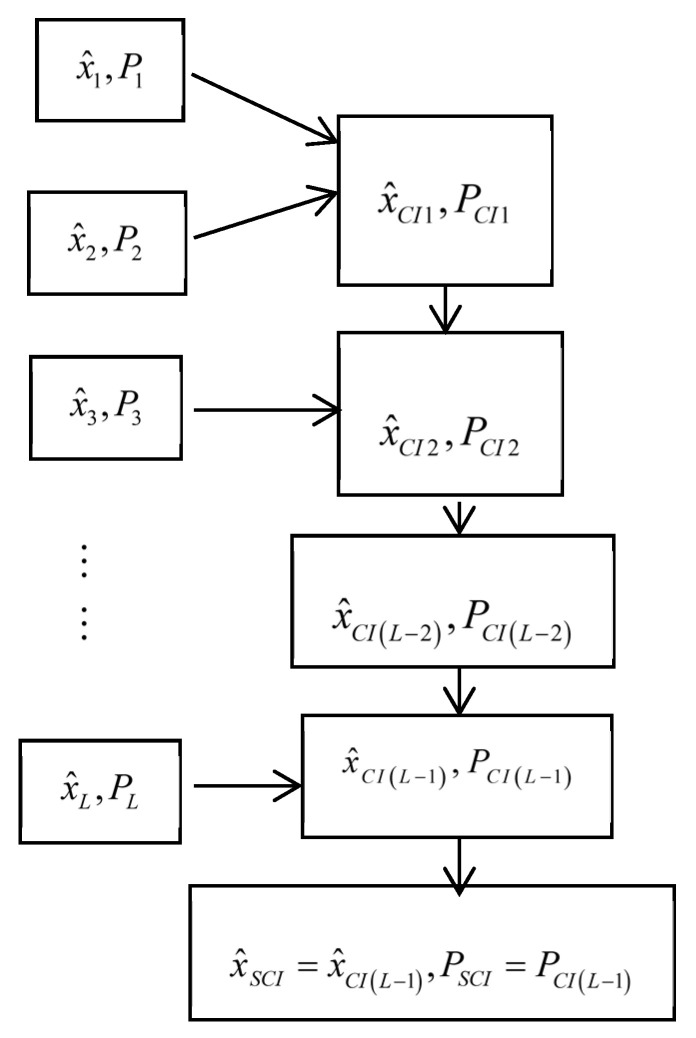
The structure of the SCI fusion robust Kalman filter.

In order to reduce the complexity and computational burden, the sequential covariance intersection (SCI) robust time-varying Kalman fuser is presented based on the L−1 two-sensor CI fused robust Kalman filters, and it can be realized by a recursive two-sensor CI fusers [34]. Its structure is shown in Figure 1, and the comparison of the computational loads of the BCI filter and the SCI filter are shown in Table 2.

Based on the two-sensor CI fused algorithm, the actual SCI fusion time-varying Kalman filter with the conservative error variances Q(t) and $R_{i}\left(t\right)$ is presented as follows

(40) $${\hat{x}}_{CIi}\left(t|t\right)=P_{CIi}\left(t|t\right)[\omega_{i}\left(t\right)P_{CI\left(i-1\right)}^{-1}\left(t|t\right){\hat{x}}_{CI\left(i-1\right)}\left(t|t\right)+\left(1-\omega_{i}\left(t\right)\right)P_{i+1}^{-1}\left(t|t\right){\hat{x}}_{i+1}^{}\left(t|t\right)]$$

(41) $$P_{CIi}\left(t|t\right)={\left[\omega_{i}\left(t\right)P_{CI\left(i-1\right)}^{-1}\left(t|t\right)+\left(1-\omega_{i}\left(t\right)\right)P_{i+1}^{-1}\left(t|t\right)\right]}^{-1},\;i=1,\cdots ,L-1$$

(42) $${\hat{x}}_{SCI}\left(t|t\right)={\hat{x}}_{CI\left(L-1\right)}\left(t|t\right),\;P_{SCI}\left(t|t\right)=P_{CI\left(L-1\right)}\left(t|t\right)$$

(43) $${\hat{x}}_{CI\left(0\right)}\left(t|t\right)={\hat{x}}_{1}\left(t|t\right),\;P_{SCI}^{-1}\left(0|0\right)=P_{1}^{-1}\left(0|0\right)$$

where ${\hat{x}}_{i}\left(t|t\right)$ are the robust local Kalman filters, and the parameters $\omega_{i}\left(t\right)$ is determined by minimizing the performance index J as

(44) $$J=\underset{\omega_{i}\left(t\right)}{\min}\mathrm{tr}P_{CIi}\left(t|t\right)=\underset{\omega_{i}\left(t\right)\in \left[0,1\right]}{\min}\mathrm{tr}\left\{{\left[\omega_{i}\left(t\right)P_{CI\left(i-1\right)}^{-1}\left(t|t\right)+\left(1-\omega_{i}\left(t\right)\right)P_{i+1}^{-1}\left(t|t\right)\right]}^{-1}\right\},\;i=1,\cdots ,L-1$$

The optimization problem (44) is equivalent to the L−1 one-dimensional optimization problems (22).

**Remark** **3.** *When the noise variances are exactly known, the optimal steady-state SCI fuser was presented in [34]. However, for multisensor systems with uncertain noise variances, the local and SCI fusion robust time-varying Kalman filters were not presented in [34].*

**Theorem** **4.** *For the multisensor uncertain system (1) and (2) with Assumptions 1–3, the actual SCI fused filter*${\hat{x}}_{SCI}\left(t|t\right)$*and its actual error variance*${\overset{¯}{P}}_{SCI}$*can be rewritten as batch representation*

(45) $${\hat{x}}_{SCI}\left(t|t\right)=P_{SCI}\left(t|t\right)\sum_{i=1}^{L}\theta_{i}^{\left(L\right)}\left(t\right)P_{i}^{-1}\left(t|t\right){\hat{x}}_{i}\left(t|t\right)\;$$

(46) $$P_{SCI}\left(t|t\right)={\left[\sum_{i=1}^{L}\theta_{i}^{\left(L\right)}\left(t\right)P_{i}^{-1}\left(t|t\right)\right]}^{-1},\;\sum_{i=1}^{L}\theta_{i}^{\left(L\right)}\left(t\right)=1,\;\theta_{i}^{\left(L\right)}\left(t\right)\geq 0\;$$

(47) $${\overset{¯}{P}}_{SCI}\left(t|t\right)=P_{SCI}\left(t|t\right)\left[\sum_{i=1}^{L}\sum_{j=1}^{L}\theta_{i}^{\left(L\right)}\left(t\right)P_{i}^{-1}\left(t|t\right){\overset{¯}{P}}_{ij}\left(t|t\right)P_{j}^{-1}\left(t|t\right)\theta_{j}^{\left(L\right)}\left(t\right)\right]P_{SCI}\left(t|t\right)\;$$

*where the weighting coefficients* $\theta_{i}^{\left(r\right)}\left(t\right)$ *can be computed recursively by*

(48) $$\theta_{i}^{\left(r\right)}\left(t\right)=\omega_{r-1}\left(t\right)\theta_{i}^{\left(r-1\right)}\left(t\right),\;i=1,\cdots ,r-1$$

(49) $$\theta_{r}^{\left(r\right)}\left(t\right)=1-\omega_{r-1}\left(t\right),\;r=2,\cdots ,L$$

(50) $$\theta_{1}^{\left(2\right)}\left(t\right)=\omega_{1}\left(t\right),\;\theta_{2}^{\left(2\right)}\left(t\right)=1-\omega_{1}\left(t\right)$$

*where the coefficients* $\omega_{i}\left(t\right)$ *are obtained by (44).*

**Proof.**  By the mathematical induction (45), (46), (48)–(50) can be proved in [32].

From (46) we have

(51) $$x\left(t\right)=P_{SCI}\left(t|t\right)\left[\sum_{i=1}^{L}\theta_{i}^{\left(L\right)}\left(t\right)P_{i}^{-1}\left(t|t\right)\right]x\left(t\right)$$

Subtracting (45) from (51), we get

(52) $${\tilde{x}}_{SCI}\left(t|t\right)=P_{SCI}\left(t|t\right)\sum_{i=1}^{L}\theta_{i}^{\left(L\right)}\left(t\right)P_{i}^{-1}\left(t|t\right){\tilde{x}}_{i}\left(t|t\right)$$

Substituting (52) into ${\overset{¯}{P}}_{SCI}\left(t|t\right)=E\left[{\tilde{x}}_{SCI}\left(t|t\right){\tilde{x}}_{SCI}^{T}\left(t|t\right)\right]$ yields the formula (47). The proof is completed. □

**Theorem** **5.** *For multisensor uncertain system (1) and (2) with Assumptions 1–3, the actual SCI fusion time-varying Kalman filter (40)–(44) is robust in the sense that for all admissible uncertainties of noise variances* $\overset{¯}{Q}\left(t\right)$ *and* ${\overset{¯}{R}}_{i}\left(t\right)$ *satisfying (4), we have*

(53) $${\overset{¯}{P}}_{SCI}\left(t|t\right)\leq P_{SCI}\left(t|t\right)\;$$

*we call (45) as the robust SCI fusion Kalman filter.*

**Proof.**  Applying Theorem 4, the SCI Kalman filter can be expressed as the equivalent BCI Kalman filter form. According to Theorem 3, the BCI time-varying fuser is robust, so that the SCI time-varying fuser is also robust. The proof is completed. □

**Remark** **4.** *The proof of Theorem 5 is different from that in [34] by the consistency of the two-sensor CI fuser. We can also prove Theorem 5 based on robustness of the two-sensor CI fuser.*

## 4. Accuracy Analysis

From (53), we can see that $P_{SCI}\left(t|t\right)$ is the upper bound of the unknown actual fused variances ${\overset{¯}{P}}_{SCI}\left(t|t\right)$ for all possible ${\overset{¯}{P}}_{i}\left(t|t\right)$ and all admissible unknown ${\overset{¯}{P}}_{ij}\left(t|t\right)$ satisfying (16), so that $P_{SCI}\left(t|t\right)$ can be viewed as the global accuracy of the SCI fuser. From (46), we see that $P_{SCI}\left(t|t\right)$ is independent of actual variances ${\overset{¯}{P}}_{i}\left(t|t\right)$ and cross-covariances ${\overset{¯}{P}}_{ij}\left(t|t\right)$. So that the global accuracy of the SCI fuser has the robustness with respect to uncertain ${\overset{¯}{P}}_{i}\left(t|t\right)$ and ${\overset{¯}{P}}_{ij}\left(t|t\right)$. From (16), we see that the uncertainties of ${\overset{¯}{P}}_{i}\left(t|t\right)$ and ${\overset{¯}{P}}_{ij}\left(t|t\right)$ are yielded by the uncertainties of $\overset{¯}{Q}\left(t\right)$ and ${\overset{¯}{R}}_{i}\left(t\right)$ satisfying (4).

**Definition** **1.** *The robustness with respect to uncertainties of noise variances of a Kalman filter is defined as its actual filtering error variances or their traces yielded by all admissible uncertainties of noise variances, which are guaranteed to have a minimal or less-conservative upper bound and this upper bound is independent of uncertainties of noise variances. The Kalman filter with robustness is called to be robust.*

**Definition** **2.** *The robust accuracy of a robust Kalman filter is defined as the trace of a minimal or less-conservative upper bound of its actual filtering error variances, while its actual accuracy is defined as the trace of its actual filtering error variance.*

**Theorem** **6.** For multisensor uncertain system (1) and (2) with Assumptions 1–3, the actual and robust accuracies of the local, BCI and SCI fused time-varying Kalman filters have the relations

(54) $$\mathrm{tr}{\overset{¯}{P}}_{i}\left(t|t\right)\leq \mathrm{tr}P_{i}\left(t|t\right),\;i=1,\cdots ,L\;$$

(55) $$\mathrm{tr}{\overset{¯}{P}}_{BCI}\left(t|t\right)\leq \mathrm{tr}P_{BCI}\left(t|t\right),\;\mathrm{tr}{\overset{¯}{P}}_{SCI}\left(t|t\right)\leq \mathrm{tr}P_{SCI}\left(t|t\right)\;$$

(56) $$\mathrm{tr}P_{BCI}\left(t|t\right)\leq \mathrm{tr}P_{i}\left(t|t\right),\;i=1,\cdots ,L\;$$

(57) $$\mathrm{tr}P_{BCI}\left(t|t\right)\leq \mathrm{tr}P_{SCI}\left(t|t\right)\;$$

(58) $$\mathrm{tr}P_{SCI}\left(t|t\right)\leq \mathrm{tr}P_{i}\left(t|t\right),\;i=1,\cdots ,L\;$$

**Proof.**  Taking the trace operations for (17), (29) and (53) yields (54) and (55). In (25), taking $\omega_{i}\left(t\right)=1$ and $\omega_{j}\left(t\right)=0$(j≠i) yields $\mathrm{tr}P_{BCI}\left(t|t\right)=\mathrm{tr}P_{i}\left(t|t\right)$, Hence, minimizing $\mathrm{tr}P_{BCI}\left(t|t\right)$ with constraints $0\leq \omega_{i}\left(t\right)\leq 1$, $\omega_{1}\left(t\right)+\cdots +\omega_{L}\left(t\right)=1$, we have $\mathrm{tr}P_{BCI}\left(t|t\right)\leq \mathrm{tr}P_{i}\left(t|t\right)$, i=1,⋯,L, i.e., (56) holds. From (45) and (46), the SCI fuser is equivalent to a BCI fuser with $\omega_{i}\left(t\right)=\theta_{i}^{\left(L\right)}\left(t\right)$, applying (25) yields (57).

The robust accuracy relation (58) can be proved by mathematical induction. For i=2, from (40)–(44) we have

(59) $${\hat{x}}_{CI1}\left(t|t\right)=P_{CI1}\left(t|t\right)\left[\omega_{1}\left(t\right)P_{1}^{-1}\left(t|t\right){\hat{x}}_{1}\left(t|t\right)+\left(1-\omega_{1}\left(t\right)\right)P_{2}^{-1}\left(t|t\right){\hat{x}}_{2}\left(t|t\right)\right]$$

(60) $$P_{CI1}\left(t|t\right)={\left[\omega_{1}\left(t\right)P_{1}^{-1}\left(t|t\right)+\left(1-\omega_{1}\left(t\right)\right)P_{2}^{-1}\left(t|t\right)\right]}^{-1},\;\omega \left(t\right)\in \left[0,1\right]$$

where ${\hat{x}}_{i}\left(t|t\right)$ are the actual local Kalman filters, the weight ω minimizes the cost function J as

(61) $$\underset{}{\min}J_{1}=\underset{\omega \left(t\right)\in \left[0,1\right]}{\min}\mathrm{tr}P_{CI1}\left(t|t\right)=\underset{\omega \in \left[0,1\right]}{\min}\mathrm{tr}\left\{{\left[\omega_{1}\left(t\right)P_{1}^{-1}\left(t|t\right)+\left(1-\omega_{1}\left(t\right)\right)P_{2}^{-1}\left(t|t\right)\right]}^{-1}\right\}$$

Taking $\omega_{1}\left(t\right)=0$, we have $J_{1}=\mathrm{tr}P_{2}\left(t|t\right)$, and taking $\omega_{1}\left(t\right)=1$, we have $J_{1}=\mathrm{tr}P_{1}\left(t|t\right)$, hence for ω(t)∈[0,1] yields

(62) $$\mathrm{tr}P_{CI1}\left(t|t\right)\leq \mathrm{tr}P_{i}\left(t|t\right),\;i=1,2$$

Similarly, for i=3, from (40)–(44) we have

(63) $$\mathrm{tr}P_{CI2}\left(t|t\right)\leq \mathrm{tr}P_{CI1}\left(t|t\right),\;\mathrm{tr}P_{CI2}\left(t|t\right)\leq \mathrm{tr}P_{3}\left(t|t\right)$$

From (62) and (63), one can obtain

(64) $$\mathrm{tr}P_{CI2}\left(t|t\right)\leq \mathrm{tr}P_{i}\left(t|t\right),\;i=1,2,3$$

By the mathematical induction method, assume that for i=L−2, the following inequality holds

(65) $$\mathrm{tr}P_{CI\left(L-2\right)}\left(t|t\right)\leq \mathrm{tr}P_{i}\left(t|t\right),\;i=1,\cdots ,L-1$$

For i=L−1, from (44), we have

(66) $$\mathrm{tr}P_{CI\left(L-1\right)}\left(t|t\right)\leq \mathrm{tr}P_{L}\left(t|t\right),\;\mathrm{tr}P_{CI\left(L-1\right)}\left(t|t\right)\leq \mathrm{tr}P_{CI\left(L-2\right)}\left(t|t\right)$$

From (65) and (66) yields

(67) $$\mathrm{tr}P_{CI\left(L-1\right)}\left(t|t\right)\leq \mathrm{tr}P_{i}\left(t|t\right),\;i=1,\cdots ,L$$

Noting that $P_{SCI}\left(t|t\right)=P_{CI\left(L-1\right)}\left(t|t\right)$, which yields the inequality (58). The proof is completed. □

**Remark** **5.** *The accuracy relations (54) and (55) mean that for all admissible uncertainties of variances satisfying (4) and (5), the actual accuracies*$\mathrm{tr}{\overset{¯}{P}}_{\theta }\left(t|t\right)$, θ=1,⋯,L,BCI,SCI*of the local or fused time-varying Kalman filter are globally controlled by*$\mathrm{tr}P_{\theta }\left(t|t\right)$, *therefore the robust accuracy*$\mathrm{tr}P_{\theta }\left(t|t\right)$*is also called the global accuracy of a robust Kalman filter. The robustness of the local and fused filters means that the robust accuracy*$\mathrm{tr}P_{\theta }\left(t|t\right)$*is independent of arbitrarily variances satisfying (4) and (5)*.

**Remark** **6.** *From the definition 2, the smaller*$\mathrm{tr}P_{\theta }\left(t|t\right)$*(or*$\mathrm{tr}{\overset{¯}{P}}_{\theta }\left(t|t\right)$*) means the higher robust (or actual) accuracy. From (54)–(58), we conclude that the robust accuracy of the robust SCI fuser is higher than that of each local robust Kalman filter, and the robust accuracy of the BCI fuser is higher than that of the SCI fuser. The actual accuracies of a robust Kalman filter are higher than its robust accuracy for all admissible uncertainties*.

**Remark** **7.** *Theorem 1 shows that*$P_{i}\left(t|t\right)$*is the minimal upper bound of*${\overset{¯}{P}}_{i}\left(t|t\right)$*in the matrix inequality sense. Theorem 3 shows that*$\mathrm{tr}P_{BCI}\left(t|t\right)$*is the minimal upper bound of*$\mathrm{tr}{\overset{¯}{P}}_{BCI}\left(t|t\right)$*in the trace inequality sense. From (55), (57) and (58) yields that*$\mathrm{tr}{\overset{¯}{P}}_{SCI}\left(t|t\right)\leq \mathrm{tr}P_{SCI}\left(t|t\right)\leq \mathrm{tr}P_{i}\left(t|t\right)$, i=1,⋯,L*so that*$\mathrm{tr}P_{SCI}\left(t|t\right)$*is a less-conservative upper bound of*$\mathrm{tr}{\overset{¯}{P}}_{SCI}\left(t|t\right)$.

## 5. Robust Local and Fused Steady-State Kalman Filters

Now we investigate the asymptotic properties of the local and fused robust time-varying Kalman filters, we shall present the corresponding steady-state robust Kalman filters. We shall also rigorously prove the convergence in a realization between the robust time-varying and steady-state Kalman filters, by the DESA method and DVESA method [37,38].

**Lemma** **1** **[39].** *Consider the following Lyapunov equation with*F*being a symmetric matrix*

(68) $$P=\Psi P\Psi^{T}+F\;$$

*where*P,Ψ*and*F*are the*n×n*matrices,*Ψ*is a stable matrix (i.e., all its eigenvalues are inside the unit circle). If*F≥0*, then*P*is symmetric and unique, and*P≥0.

**Lemma** **2** **[38].** *Consider the time-varying Lyapunov equation*

(69) $$P\left(t\right)=F_{1}\left(t\right)P\left(t-1\right)F_{2}{}^{T}\left(t\right)+U\left(t\right)\;$$

*where *t≥0*, the output*P(t) *and the input* U(t) *are the* n×n *matrices, and the* n×n *matrices* $F_{1}\left(t\right)$ *and* $F_{2}\left(t\right)$ *are uniformly asymptotically stable, i.e., there exist constants* $0<\rho_{j}<1$ *and* $c_{j}>0$ *such that*

(70) $$\left\|F_{j}\left(t,i\right)\right\|\leq c_{j}\rho_{j}^{t-i},\forall t\geq i\geq 0,\;j=1,2$$

*If* U(t)*is bounded, then*P(t)*is bounded. If*U(t)→0*, then*P(t)→0*, as*t→∞*. Notice that*U(t)*is called to be bounded, if*‖U(t)‖≤c*(constant), for arbitrary*t≥0.

**Lemma** **3** **[37].** *Consider a dynamic error system*

(71) δ(t)=F(t)δ(t−1)+u(t) 

*where*$\delta \left(t\right)\in R^{n}$*,*$u\left(t\right)\in R^{n}$*, and*F(t)*is uniformly asymptotically stable. If*u(t)*is bounded, then*δ(t)*is bounded. If*u(t)→0*, then*δ(t)→0*, as*t→∞.

**Theorem** **7.** *For multisensor uncertain time-invariant system (1) and (2) with Assumptions 1–2, where*Φ(t)=Φ, Γ(t)=Γ, $H_{i}\left(t\right)=H_{i}$, $Q\left(t\right)=Q,R_{i}\left(t\right)=R_{i}$, $\overset{¯}{Q}\left(t\right)=\overset{¯}{Q}$*and*${\overset{¯}{R}}_{i}\left(t\right)={\overset{¯}{R}}_{i}$*are all the constant matrices. If each subsystem with conservative noise variances*Q*and*$R_{i}$*is completely observable and completely controllable, then the actual local steady-state Kalman filters are given as*

(72) $${\hat{x}}_{i}^{s}\left(t|t\right)=\Psi_{i}{\hat{x}}_{i}^{s}\left(t-1|t-1\right)+K_{i}y_{i}\left(t\right),\;i=1,\cdots ,L\;$$

(73) $$\Psi_{i}=\left[I_{n}-K_{i}H_{i}\right]\Phi ,\;K_{i}=\Sigma_{i}H_{i}^{T}{\left(H_{i}\Sigma_{i}H_{i}^{T}+R_{i}\right)}^{-1}\;$$

(74) $$P_{i}=\left[I_{n}-K_{i}H_{i}\right]\Sigma_{i}\;$$

*where* $y_{i}\left(t\right)$ *are the actual measurements, and the initial value* ${\hat{x}}_{i}^{s}\left(0|0\right)$ *can arbitrarily be selected.* $\Sigma_{i}$ *satisfies the steady-state Riccati equations*

(75) $$\Sigma_{i}=\Phi \left[\Sigma_{i}-\Sigma_{i}H_{i}^{T}{\left(H_{i}\Sigma_{i}H_{i}^{T}+R_{i}\right)}^{-1}H_{i}\Sigma_{i}\right]\Phi^{T}+\Gamma Q\Gamma^{T}$$

*and the conservative cross-covariances*$P_{ij}$ *and the actual cross-covariances*${\overset{¯}{P}}_{ij}$*satisfy the steady-state Lyapunov equations*

(76) $$P_{ij}=\Psi_{i}P_{ij}\Psi_{j}^{T}+\left[{Ι}_{n}-{Κ}_{i}H_{i}\right]\Gamma Q\Gamma^{T}{\left[I_{n}-K_{j}H_{j}\right]}^{T}+K_{i}R_{i}K_{j}^{T}\delta_{ij},i,j=1,\cdots ,L$$

(77) $${\overset{¯}{P}}_{ij}=\Psi_{i}{\overset{¯}{P}}_{ij}\Psi_{j}^{T}+\left[{Ι}_{n}-{Κ}_{i}H_{i}\right]\Gamma \overset{¯}{Q}\Gamma^{T}{\left[I_{n}-K_{j}H_{j}\right]}^{T}+K_{i}{\overset{¯}{R}}_{i}K_{j}^{T}\delta_{ij},i,j=1,\cdots ,L$$

*with the definition* $P_{i}=P_{ii}$*,*${\overset{¯}{P}}_{i}={\overset{¯}{P}}_{ii}$*, and we have*

(78) $$P_{ij}\left(t|t\right)\to P_{ij},\;\mathrm{as}\;t\to \infty ,\;i,j=1,\cdots ,L$$

(79) $${\overset{¯}{P}}_{ij}\left(t|t\right)\to {\overset{¯}{P}}_{ij},\;\mathrm{as}\;t\to \infty ,\;i,j=1,\cdots ,L$$

*The actual local steady-state Kalman filters (72) are robust in the sense that for all admissible uncertainties of* $\overset{¯}{Q}$ *and* ${\overset{¯}{R}}_{i}$ *satisfying* $\overset{¯}{Q}\leq Q,\;{\overset{¯}{R}}_{i}\leq R_{i}$*, then*

(80) $${\overset{¯}{P}}_{i}\leq P_{i},\;i=1,\cdots ,L$$

*and* $P_{i}$ *is the minimal upper bound of* ${\overset{¯}{P}}_{i}$ *. They are called the robust local steady-state Kalman filters.*

**Proof.**  According to the complete observability and complete controllability of each subsystem, we have [40]

(81) $$P_{i}\left(t|t-1\right)\to \Sigma_{i},\;\mathrm{as}t\to \infty ,\;i=1,\cdots ,L$$

Then from (7), (8) and (11), we have

(82) $$\Psi_{i}\left(t\right)\to \Psi_{i},\;K_{i}\left(t\right)\to K_{i},P_{i}\left(t|t\right)\to P_{i},\;\mathrm{as}\;t\to \infty ,i=1,\cdots ,L$$

where $\Psi_{i}$ are stable matrices [40], and $\Psi_{i}\left(t\right)$ are uniformly asymptotically stable [40]. When t→∞, taking the limit operations for (6)–(11), (12) and (16), we obtain (72)–(77). From $K_{i}\left(t\right)\to K_{i}$, the gains $K_{i}\left(t\right)$ are bounded, which yields the boundedness of the input of the Lyapunov Equation (12). Hence, applying Lemma 2 to (12) yields that $P_{ij}\left(t|t\right)$ are bounded. Setting $\Psi_{i}\left(t\right)=\Psi_{i}+\Delta \Psi_{i}\left(t\right)$ with $\Delta \Psi_{i}\left(t\right)\to 0$, and subtracting (76) from (12) with $H_{i}\left(t\right)=H_{i},\;\Gamma \left(t\right)=\Gamma ,\;Q\left(t\right)=Q$ and $R_{i}\left(t\right)=R_{i}$, and defining $\Delta_{ij}\left(t\right)=P_{ij}\left(t|t\right)-P_{ij}$, yields the Lyapunov equations

(83) $$\Delta_{ij}\left(t\right)=\Psi_{i}\Delta_{i}\left(t-1\right)\Psi_{j}^{T}+U_{ij}\left(t\right)$$

(84) $$\begin{array}{l} U_{ij}\left(t\right)=\left[{Ι}_{n}-{Κ}_{i}\left(t\right)H_{i}\right]\Gamma Q\Gamma^{T}{\left[I_{n}-K_{j}\left(t\right)H_{j}\right]}^{T}+K_{i}\left(t\right)R_{i}K_{j}^{T}\left(t\right)\delta_{ij}\\ -\left[{Ι}_{n}-{Κ}_{i}H_{i}\right]\Gamma Q\Gamma^{T}\left[I_{n}-K_{j}H_{j}\right]-K_{i}R_{i}K_{j}^{T}\delta_{ij}+\Psi_{i}P_{ij}\left(t-1|t-1\right)\Delta \Psi_{j}^{T}\left(t\right)\\ +\Delta \Psi_{i}\left(t\right)P_{ij}\left(t-1|t-1\right)\Psi_{j}^{}+\Delta \Psi_{i}\left(t\right)\Delta \Psi_{j}^{T}\left(t\right) \end{array}$$

Applying $K_{i}\left(t\right)\to K_{i}$, the boundedness of $P_{ij}\left(t|t\right)$, and $\Delta \Psi_{i}\left(t\right)\to 0$ yields that $U_{ij}\left(t\right)\to 0$. Applying Lemma 2 to (83) yields $\Delta_{ij}\left(t\right)\to 0$, as t→∞, i.e., (78) holds. Similarly, we can prove (79). Taking the limit operation for (17), as t→∞, and applying (78) and (79) yields (80). Taking $\overset{¯}{Q}=Q,\;{\overset{¯}{R}}_{i}=R_{i}$, subtracting (77) from (76), and applying Lemma 1 yields ${\overset{¯}{P}}_{i}=P_{i}$, if $P_{i}^{*}$ is arbitrary other upper bound of ${\overset{¯}{P}}_{i}$ for all admissible $\overset{¯}{Q}$ and ${\overset{¯}{R}}_{i}$ satisfying $\overset{¯}{Q}\leq Q,\;{\overset{¯}{R}}_{i}\leq R_{i}$, then we have $P_{i}={\overset{¯}{P}}_{i}\leq P_{i}^{*}$, which yields that $P_{i}$ is the minimal. The proof is completed. □

**Theorem** **8.** *For multisensor uncertain time-invariant system (1) and (2) with Assumptions 1–2, if each subsystem with conservative noise variances*Q*and*$R_{i}$*is completely observable and completely controllable, then the actual steady-state BCI fusion Kalman filter is given as*

(85) $${\hat{x}}_{BCI}^{s}\left(t|t\right)=P_{BCI}\sum_{i=1}^{L}\omega_{i}P_{i}^{-1}{\hat{x}}_{i}^{s}\left(t|t\right)\;$$

(86) $$P_{BCI}={\left[\sum_{i=1}^{L}\omega_{i}P_{i}^{-1}\right]}^{-1}$$

*where* ${\hat{x}}_{i}^{s}\left(t|t\right)$ *are given in Theorem 7, and the optimal weighting coefficients* $\omega_{i}$ *are obtained by minimizing the performance index* $J=\mathrm{tr}P_{BCI}$ *as*

(87) $$\min J=\underset{\omega_{i}}{\min}\mathrm{tr}P_{BCI}=\underset{\begin{array}{l} \omega_{i}\in \left[0,1\right]\\ \omega_{1}+\cdots +\omega_{L}=1 \end{array}}{\min}\mathrm{tr}\left\{{\left[\sum_{i=1}^{L}\omega_{i}P_{i}^{-1}\right]}^{-1}\right\}$$

*It has the robustness in the sense that for all admissible uncertainties of* $\overset{¯}{Q}$ *and* ${\overset{¯}{R}}_{i}$ *satisfying* $\overset{¯}{Q}\leq Q,\;{\overset{¯}{R}}_{i}\leq R_{i}$*, we have*

(88) $${\overset{¯}{P}}_{BCI}\leq P_{BCI}$$

*where the actual fused steady-state filtering error covariance is given as*

(89) $${\overset{¯}{P}}_{BCI}=P_{BCI}\left[\sum_{i=1}^{L}\sum_{j=1}^{L}\omega_{i}P_{i}^{-1}{\overset{¯}{P}}_{ij}P_{j}^{-1}\omega_{j}\right]P_{BCI}$$

*and* $\mathrm{tr}P_{BCI}$ *is the minimal upper bound of* $\mathrm{tr}{\overset{¯}{P}}_{BCI}$ *. It is called the robust steady-state BCI fusion Kalman filter.*

**Proof.**  As t→∞, taking the limit operations for (23)–(26) yields (85)–(87). Taking the limit operations for (24) and (26) and applying (78) and (79) yields that $P_{SCI}\left(t|t\right)\to P_{SCI},\;{\overset{¯}{P}}_{SCI}\left(t|t\right)\to {\overset{¯}{P}}_{SCI}$, so that taking the limit operations for (26) and (29) yields (88) and (89). The proof is completed. □

**Theorem** **9.** *For multisensor uncertain time-invariant system (1) and (2) with Assumptions 1–2, if each subsystem with conservative noise variances*Q*and*$R_{i}$*is completely observable and completely controllable, the actual steady-state SCI fusion Kalman filter is given as*

(90) $${\hat{x}}_{SCI}^{s}\left(t|t\right)=P_{SCI}\sum_{i=1}^{L}\theta_{i}^{\left(L\right)}P_{i}^{-1}{\hat{x}}_{i}^{s}\left(t|t\right)\;$$

(91) $$P_{SCI}={\left[\sum_{i=1}^{L}\theta_{i}^{\left(L\right)}P_{i}^{-1}\right]}^{-1},\;\sum_{i=1}^{L}\theta_{i}^{\left(L\right)}=1,\;\theta_{i}^{\left(L\right)}\geq 0\;$$

(92) $${\overset{¯}{P}}_{SCI}=P_{SCI}\left[\sum_{i=1}^{L}\sum_{j=1}^{L}\theta_{i}^{\left(L\right)}P_{i}^{-1}{\overset{¯}{P}}_{ij}P_{j}^{-1}\theta_{j}^{\left(L\right)}\right]P_{SCI}\;$$

*where the weighting coefficients* $\theta_{i}^{\left(r\right)}$ *can be computed recursively by*

(93) $$\theta_{i}^{\left(r\right)}=\omega_{r-1}\theta_{i}^{\left(r-1\right)},\;i=1,\cdots ,r-1$$

(94) $$\theta_{r}^{\left(r\right)}=1-\omega_{r-1},\;r=2,\cdots ,L$$

(95) $$\theta_{1}^{\left(2\right)}=\omega_{1},\;\theta_{2}^{\left(2\right)}=1-\omega_{1}$$

*and it is robust in the sense that for all admissible uncertainties*$\overset{¯}{Q}$*and*${\overset{¯}{R}}_{i}$*satisfying* $\overset{¯}{Q}\leq Q,\;{\overset{¯}{R}}_{i}\leq R_{i}$*, we have*

(96) $${\overset{¯}{P}}_{SCI}\leq P_{SCI}$$

*It is called the robust steady-state SCI fusion Kalman filter.*

**Proof.**  As t→∞, taking the limit operations for (45)–(47), and (53) yields (90)–(92), and (96). From (48)–(50), we have (93)–(95). The proof is completed. □

**Theorem** **10.** *Under the conditions of Theorem 7, if the measurement data of* $y_{i}\left(t\right)$ *are bounded, then the robust local time-varying and steady-state Kalman filters* ${\hat{x}}_{i}^{}\left(t|t\right)$ *and* ${\hat{x}}_{i}^{s}\left(t|t\right)$ *given by (6) and (72) have each other the convergence in a realization, such that*

(97) $$\left[{\hat{x}}_{i}\left(t|t\right)-{\hat{x}}_{i}^{s}\left(t|t\right)\right]\to 0,\;\mathrm{as},\;i.a.r\;$$

**Proof.**  Setting $\Psi_{i}\left(t\right)=\Psi_{i}+\Delta \Psi_{i}\left(t\right)$, $K_{i}\left(t\right)=K_{i}+\Delta K_{i}\left(t\right)$ in (6), applying (82) yields $\Delta \Psi_{i}\left(t\right)\to 0$, $\Delta K_{i}\left(t\right)\to 0$, as t→∞. Subtracting (72) from (6), and defining $\delta_{i}\left(t\right)={\hat{x}}_{i}\left(t|t\right)-{\hat{x}}_{i}^{s}\left(t|t\right)$, we have

(98) $$\delta_{i}\left(t\right)=\Psi_{i}\delta_{i}\left(t-1\right)+u_{i}\left(t\right)$$

with $u_{i}\left(t\right)=\Delta \Psi_{i}\left(t\right){\hat{x}}_{i}\left(t-1|t-1\right)+\Delta K_{i}\left(t\right)y_{i}\left(t\right)$. Noting that $\Psi_{i}\left(t\right)$ is uniformly asymptotically stable, and $\Delta K_{i}\left(t\right)y_{i}\left(t\right)$ is bounded, applying Lemma 3 to (6) yields the boundedness of ${\hat{x}}_{i}^{}\left(t|t\right)$. Hence, we have $u_{i}\left(t\right)\to 0$. Applying Lemma 3 to (98), noting that $\Psi_{i}$ is a stable matrix, so it is also uniformly asymptotically stable, hence $\delta_{i}\left(t\right)\to 0$, i.e., the convergence (97) holds. The proof is completed. □

**Theorem** **11.** *Under the conditions of Theorem 10, the robust time-varying and steady-state SCI fusers* ${\hat{x}}_{SCI}^{}\left(t|t\right)$ *and* ${\hat{x}}_{SCI}^{s}\left(t|t\right)$ *have each other the convergence in a realization, such that*

(99) $$\left[{\hat{x}}_{SCI}\left(t|t\right)-{\hat{x}}_{SCI}^{s}\left(t|t\right)\right]\to 0,\;\mathrm{as}\;t\to \infty ,\;i.a.r\;$$

**Proof.**  From (87), the minimal value point $(\omega_{1},\cdots ,\omega_{L})\in R^{L}$ of $J=\mathrm{tr}P_{BCI}$ is obtained by solving nonlinear equations

(100) $$\frac{\partial J}{\partial \omega_{1}}=0,\cdots ,\frac{\partial J}{\partial \omega_{L}}=0$$

According to the existence theorem [36] of implicit function, in a sufficiently small neighborhood of the point $(P_{i}^{ks},i=1,\cdots ,L,,s=1,\cdots ,n)\in R^{Ln^{2}}$ with the definition $P_{i}=(P_{i}^{ks})$, k,s=1,⋯,n, $\omega_{i}$ can be represented by a $Ln^{2}$-dimension continuous function of all elements of $P_{i}(i=1,\cdots ,L)$ as

(101) $$\omega_{i}=f_{i}(P_{1},\cdots ,P_{L}),\;i=1,\cdots ,L$$

Applying (78) with i=j yields $P_{i}\left(t|t\right)\to P_{i}$, as t→∞. Hence for sufficiently larger t, we have

(102) $$\omega_{i}(t)=f_{i}(P_{1}(t|t),\cdots ,P_{L}(t|t)),\;i=1,\cdots ,L$$

where $\omega_{i}(t)$ are defined in (25). According to the continuity of $f_{i}$, if follows

(103) $$\omega_{i}(t)\to \omega_{i},\;\mathrm{as}\;t\to \infty ,\;i=1,\cdots ,L$$

and applying (48)–(50) and (93)–(95) yields

(104) $$\theta_{i}^{\left(L\right)}(t)\to \theta_{i}^{L},\;\mathrm{as}\;t\to \infty ,\;i=1,\cdots ,L$$

Defining

(105) $$\Omega_{i}=P_{SCI}\theta_{i}^{\left(L\right)}P_{i}^{-1},\;\Omega_{i}(t)=P_{SCI}(t|t)\theta_{i}^{\left(L\right)}(t)P_{i}^{-1}(t|t)=\Omega_{i}+\Delta \Omega_{i}(t)$$

Applying (78) with i=j, (46), (91) and (104) yields $\Omega_{i}(t)\to \Omega_{i}$, as t→∞, which yields $\Delta \Omega_{i}(t)\to 0$.

Subtracting (85) from (45) and applying (105) yields

(106) $${\hat{x}}_{SCI}(t|t)-{\hat{x}}_{SCI}^{s}(t|t)=\sum_{i=1}^{L}\Omega_{i}({\hat{x}}_{i}(t|t)-{\hat{x}}_{i}^{s}(t|t))+\sum_{i=1}^{L}\Delta \Omega_{i}(t){\hat{x}}_{i}(t|t)\;$$

Applying (82) yields the boundedness of $K_{i}(t)$, and applying the boundedness of $y_{i}(t)$ yields that $K_{i}(t)y_{i}(t)$ is bounded. Noting that $\Psi_{i}(t)$ is uniformly asymptotically stable [40]. Applying Lemma 3 to (6) yields that ${\hat{x}}_{i}(t|t)$ is bounded. Hence applying (97), (106) and $\Delta \Omega_{i}(t)\to 0$ yields (99). The proof is completed. □

**Theorem** **12.** *Under the conditions of Theorem 10, the robust accuracy comparison of the local and the fused robust steady-state Kalman filters is given by*

(107) $$\mathrm{tr}{\overset{¯}{P}}_{i}\leq \mathrm{tr}P_{i},\;i=1,\cdots ,L,\;\mathrm{tr}{\overset{¯}{P}}_{BCI}\leq \mathrm{tr}P_{BCI},\;\mathrm{tr}{\overset{¯}{P}}_{SCI}\leq \mathrm{tr}P_{SCI}\;$$

(108) $$\mathrm{tr}P_{BCI}\leq \mathrm{tr}P_{i},\;i=1,\cdots ,L\;$$

(109) $$\mathrm{tr}P_{BCI}\leq \mathrm{tr}P_{SCI}\;$$

(110) $$\mathrm{tr}P_{SCI}\leq \mathrm{tr}P_{i},\;i=1,\cdots ,L\;$$

**Proof.**  Applying (78), (79), (103) and (104) yields that ${\overset{¯}{P}}_{i}\left(t|t\right)\to P_{i},{\overset{¯}{P}}_{BCI}\left(t|t\right)\to P_{BCI},$ ${\overset{¯}{P}}_{SCI}\left(t|t\right)\to P_{SCI}$. As t→∞, taking the limit operations for (54)–(58) yields Theorem 12. The proof is completed. □

## 6. Sensitivity Problem

**Figure 2:**
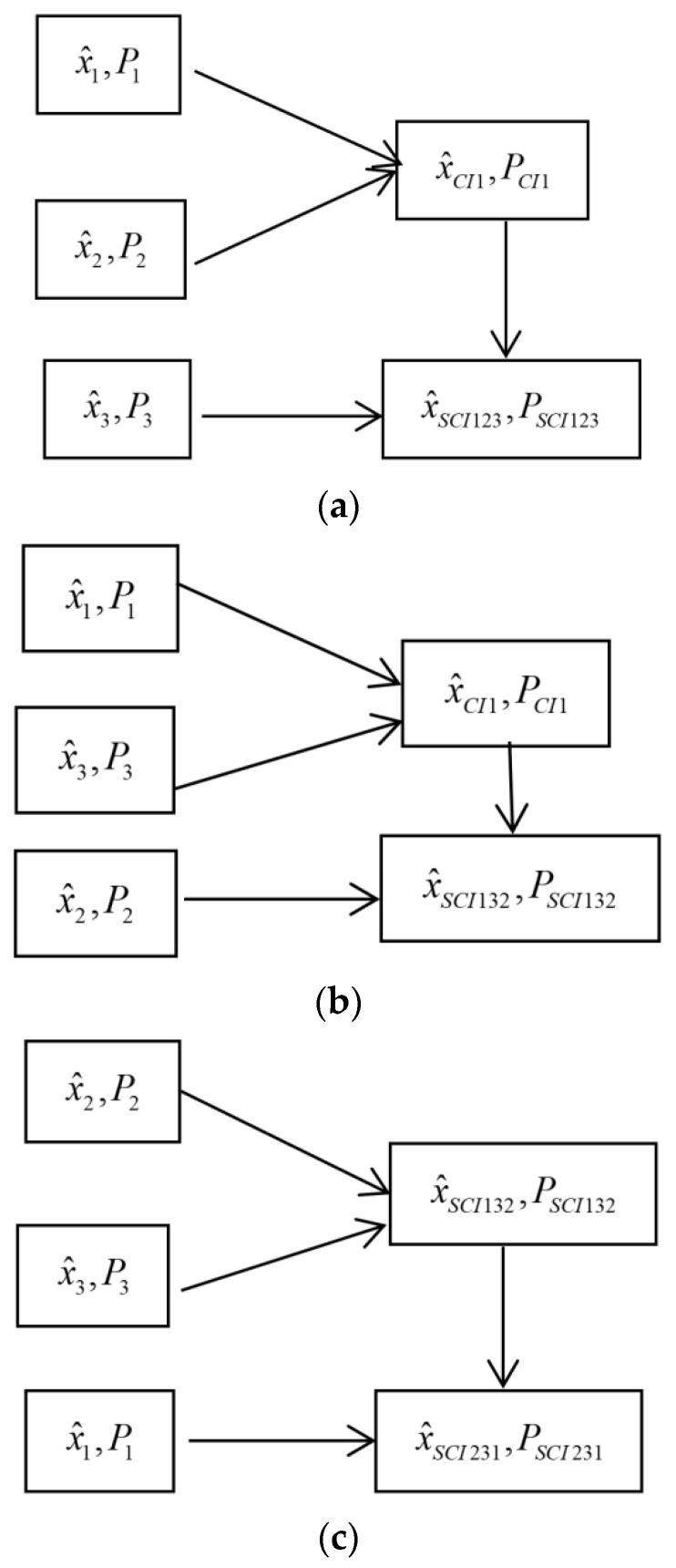
The fused orders of the SCI fusers in the L=3 case. (**a**) The order 1: SCI123; (**b**) The order 2: SCI132; (**c**) The order 3: SCI231.

For the SCI fusion robust Kalman filter, the fused schemes are different with respect to different orders of sensors. For example, in the case where there are three fused structures as shown in Figure 2, the problem is that whether the SCI fused robust accuracy is sensitive with respect to the fused orders of sensors. The following two sensor simulation examples will show that the robust accuracy of the SCI fuser is not very sensitive with respect to the orders of the sensors.

## 7. Simulation Examples

**Example** **1.** *Consider a 3-sensor tracking system with uncertain noise variances*

(111) x(t+1)=Φx(t)+Γw(t) 

(112) $$y_{i}\left(t\right)=H_{i}x\left(t\right)+v_{i}\left(t\right),\;i=1,2,3\;$$

(113) $$\Phi =\left[\begin{array}{cc} 1 & T_{0}\\ 0 & 1 \end{array}\right],\;\Gamma =\left[\begin{array}{c} 0.5{Τ}_{0}^{2}\\ T_{0} \end{array}\right],\;H_{1}=\left[\begin{array}{cc} 1 & 0 \end{array}\right],\;H_{2}=I_{2},\;H_{3}=\left[\begin{array}{cc} 1 & 0 \end{array}\right]\;$$

*where*$T_{0}=0.25$*is the sampled period,*$x\left(t\right)={\left[x_{1}\left(t\right),x_{2}\left(t\right)\right]}^{T}$*is the state,*$x_{1}\left(t\right)$*and*$x_{2}\left(t\right)$*are the position and velocity of target at time*$tT_{0}$. $y_{i}\left(t\right)$*is the measurement,*w(t)*and*$v_{i}\left(t\right)$*are independent Gaussion white noises with zero mean and unknown variances*$\overset{¯}{Q}$*and*${\overset{¯}{R}}_{i}$, *respectively,*Q*and*$R_{i}$*are conservative upper bounds of*$\overset{¯}{Q}$*and*${\overset{¯}{R}}_{i}$*satisfying*$\overset{¯}{Q}\leq Q,\;{\overset{¯}{R}}_{i}\leq R_{i}$*. In the simulation, we take* Q=1*,* $R_{1}=0.8$*,* $R_{2}=diag(8,0.36)$*,*$R_{3}=0.5$*,*$\overset{¯}{Q}=0.8$*,*${\overset{¯}{R}}_{1}=0.65$*,*${\overset{¯}{R}}_{2}=diag(6,0.25)$*,*${\overset{¯}{R}}_{3}=0.45$.

**Figure 3:**
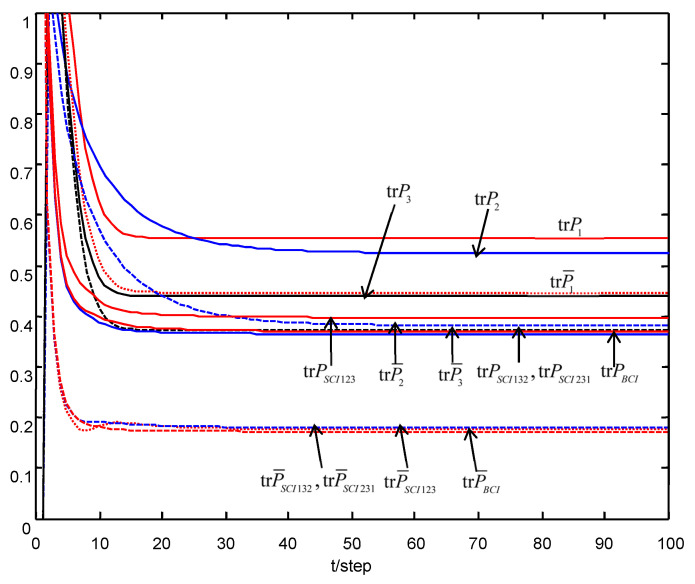
The robust accuracy relations of the local and fused robust Kalman filters.

The traces of the conservative and actual local robust filtering error variances are compared in Figure 3. For Figure 3, we see that the traces of the local and fused robust time-varying Kalman filters quickly converge to these of the corresponding steady-state Kalman filters, which verify the robust accuracy relations (54)–(58), and their steady-state robust and actual accuracy relations (107)–(110).

The robust and actual accuracy comparisons are shown in Table 3 and Table 4. From Table 3 and Table 4, we see that the SCI fused robust accuracy $\mathrm{tr}P_{SCI123}$, $\mathrm{tr}P_{SCI132}$ and $\mathrm{tr}P_{SCI321}$ are close or equal to the BCI fused robust accuracy $\mathrm{tr}P_{BCI}$, and the accuracy of the SCI fuser is not very sensitive with respect to the orders of sensor. We also see that the actual accuracy of the SCI fuser, and $\mathrm{tr}{\overset{¯}{P}}_{SCI123}$, $\mathrm{tr}{\overset{¯}{P}}_{SCI132}$ and $\mathrm{tr}{\overset{¯}{P}}_{SCI321}$ are close to or equal to the actual accuracy of the BCI fuser $\mathrm{tr}{\overset{¯}{P}}_{BCI}$; they are all higher than the robust accuracy of each local filter, which verify the accuracy relations (54)–(58) and their steady-state robust and actual accuracy relations (107)–(110).

**Figure 4:**
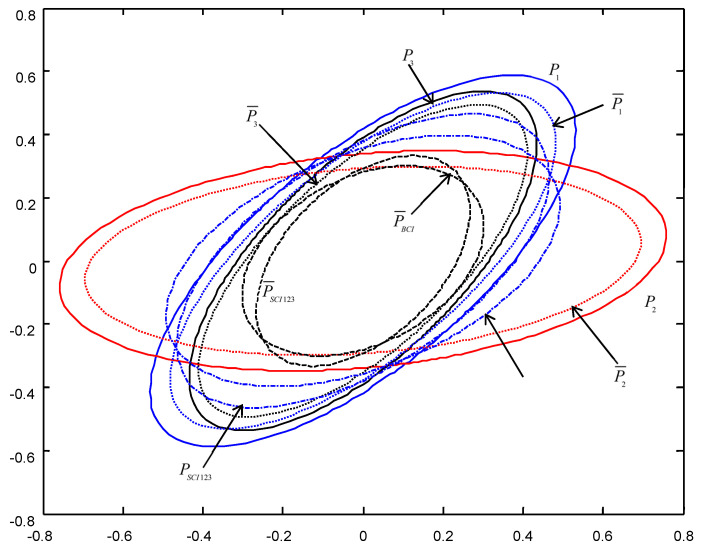
The ellipses of the actual and conservative time-varying filtering error variances of the order SCI123 at t=10.

**Figure 5:**
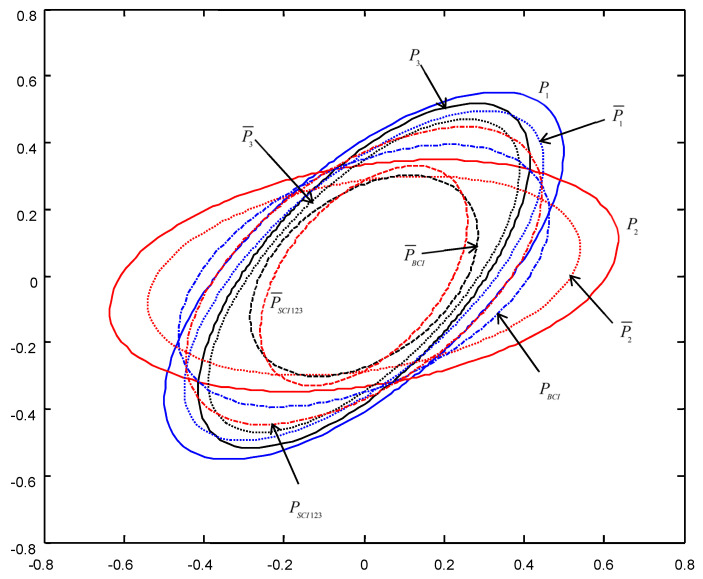
The ellipses of the actual and conservative steady-state filtering error variances of the order SCI123.

**Figure 6:**
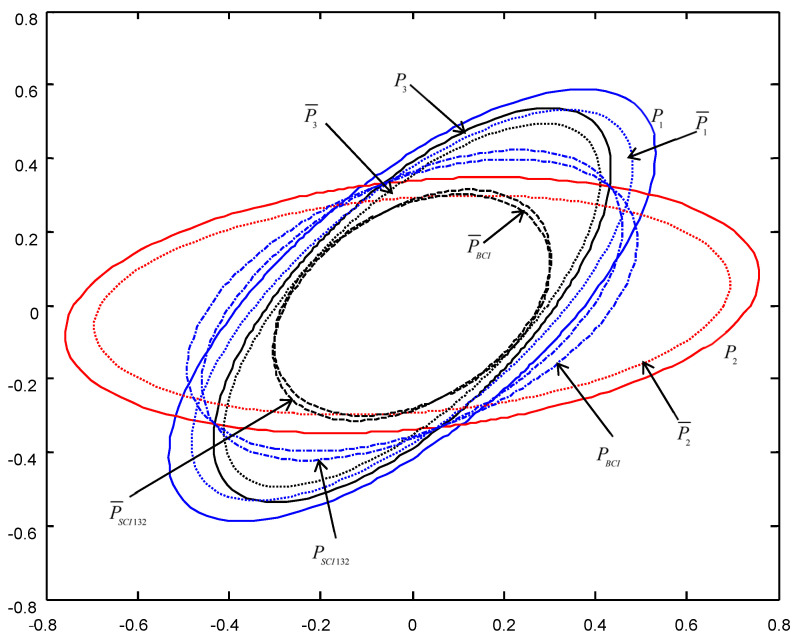
The ellipses of the actual and conservative time-varying filtering error variances of the order SCI132 at t=10.

**Figure 7:**
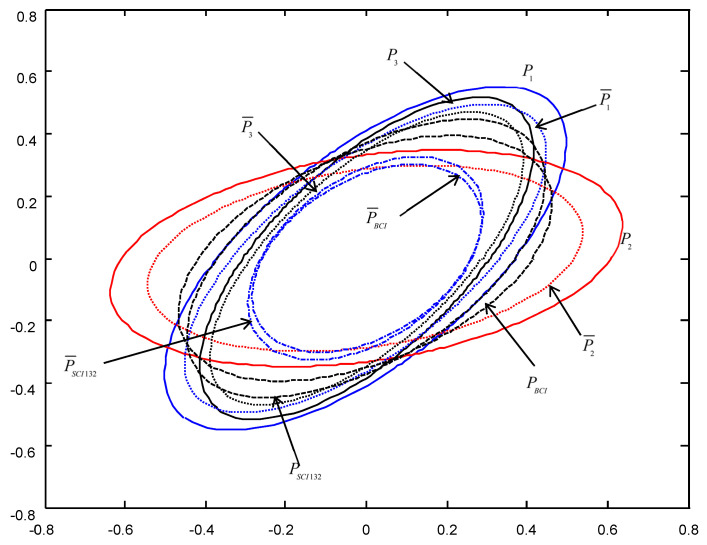
The ellipses of the actual and conservative steady-state filtering error variances of the order SCI132.

**Figure 8:**
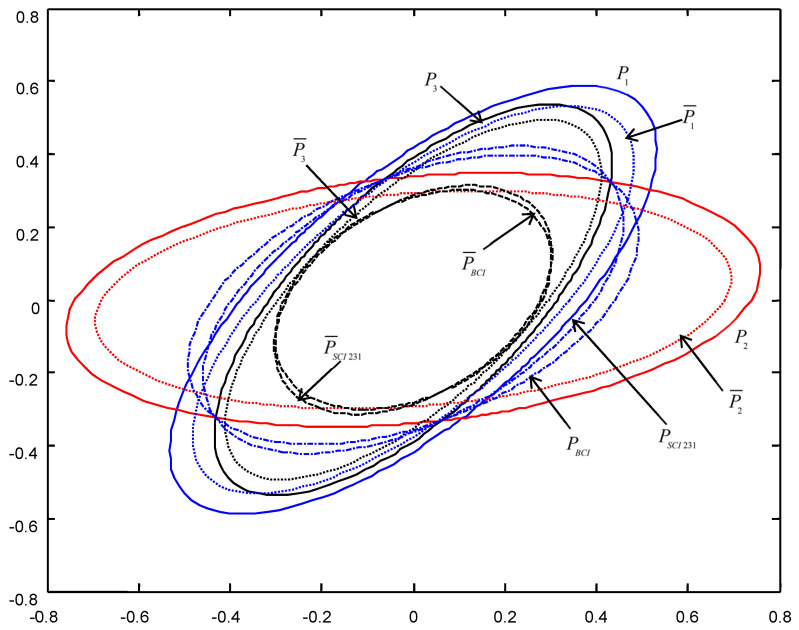
The ellipses of the actual and conservative time-varying filtering error variances of the order SCI231 at t=10.

**Figure 9:**
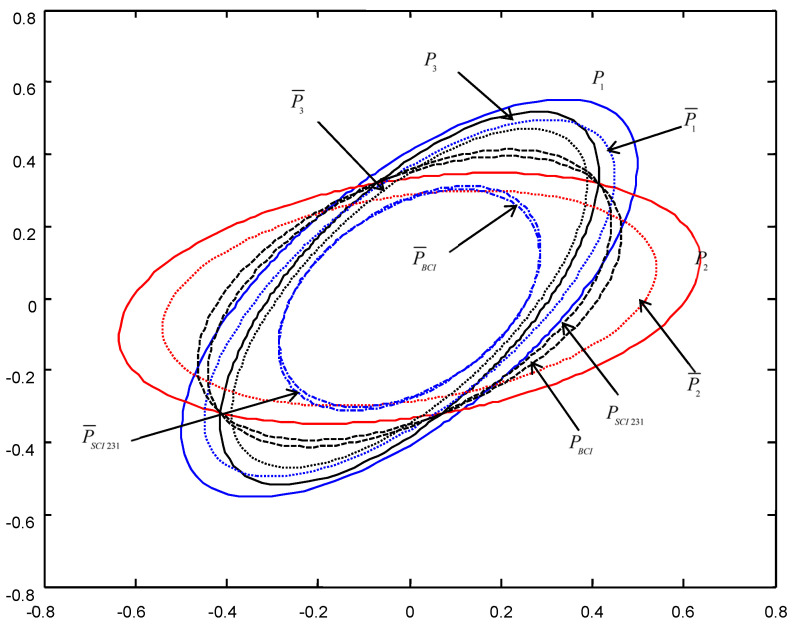
The ellipses of the actual and conservative steady-state filtering error variances of the order SCI231.

In order to give a geometric interpretation of the accuracy relations, The covariance ellipses of the robust time-varying Kalman filters at time t=10 and robust steady-state Kalman filters are shown in Figure 4, Figure 5, Figure 6, Figure 7, Figure 8 and Figure 9.

From Figure 4, Figure 5, Figure 6, Figure 7, Figure 8 and Figure 9, we see that the ellipses of the actual variances ${\overset{¯}{P}}_{i}$(i=1,2,3) are all enclosed in that of the conservative variances $P_{i}$, respectively, which verify the robustness (17). The ellipses of actual BCI and SCI fused variances ${\overset{¯}{P}}_{BCI}$ and ${\overset{¯}{P}}_{SCIijk}$ (ijk=123,132,231) are respectively enclosed in those of $P_{BCI}$ and $P_{SCIijk}$, which verifies the robustness (29) and (53). Moreover, we see that the ellipse of ${\overset{¯}{P}}_{BCI}$ is close to or equal to that of ${\overset{¯}{P}}_{SCIijk}$, the ellipse of $P_{BCI}$ is close to or equal to that of $P_{SCIijk}$, which means that the robust accuracies of the SCI fusers with different orders of sensors are close to those of the BCI fusers, and the robust and actual accuracies of the SCI fusers are not very sensitive to the orders of sensors.

**Figure 10:**
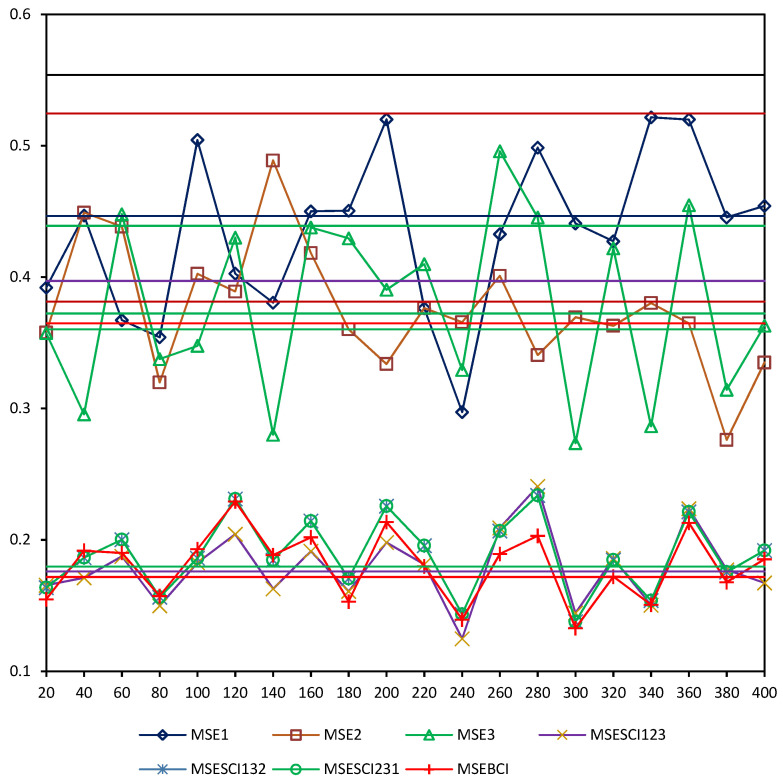
The comparison of ${\mathrm{MSE}}_{i}\left(t\right)$ and $\mathrm{tr}P_{i}$, i=1,2,3,SCI123,SCI132,SCI231,BCI.

In order to verify the above theoretical accuracy relations, taking N=200 runs, the mean square error (MSE) value at time t of the local and fused robust Kalman filters are shown in Figure 10. From Figure 10, we see that when t is sufficiently large, we have the accuracy relations

(114) $${\mathrm{MSE}}_{\theta }\left(t\right)\leq \mathrm{tr}P_{\theta },\;\theta =1,2,3,BCI,SCI$$

and the curves of ${\mathrm{MSE}}_{\theta }\left(t\right)$ are close to the straight lines corresponding to $\mathrm{tr}{\overset{¯}{P}}_{\theta }$, which verify the robust accuracy relations (107) and the robust accuracy relations in Table 3.

**Example** **2.** *In order to show the sensitivity of the actual and robust accuracies for the SCI fuser with respect to the orders of sensors, consider a 4-sensor tracking system with uncertainties of noise variances*

(115) x(t+1)=Φx(t)+Γw(t) 

(116) $$y_{i}\left(t\right)=H_{i}x\left(t\right)+v_{i}\left(t\right),\;i=1,2,3,4\;$$

(117) $$\Phi =\left[\begin{array}{cc} 1 & T_{0}\\ 0 & 1 \end{array}\right],\;\Gamma =\left[\begin{array}{c} 0.5{Τ}_{0}^{2}\\ T_{0} \end{array}\right],\;H_{1}=\left[\begin{array}{cc} 1 & 0 \end{array}\right],\;H_{2}=I_{2},\;H_{3}=\left[\begin{array}{cc} 1 & 0 \end{array}\right],\;H_{4}=I_{2}\;$$

In the simulation,

$$\;\;\;\;\;\;\;\;\;\;\;T_{0}=0.25,\;Q=1,\;R_{1}=0.8,\;R_{2}=diag(8,0.36),\;R_{3}=0.5,\;R_{4}=diag(0.25,10),\;\overset{¯}{Q}=0.8,\;\;{\overset{¯}{R}}_{1}=0.65,\;{\overset{¯}{R}}_{2}=diag(6,0.25),\;{\overset{¯}{R}}_{3}=0.45,\;{\overset{¯}{R}}_{4}=diag(0.2,9).$$

Similar to Figure 3, for the sensor number L=4, there are 12 fused orders as follows:

*SCI*1234, *SCI*1243, *SCI*1324, *SCI*1342, *SCI*1423, *SCI*1432, *SCI*2314, *SCI*2341, *SCI*2413, *SCI*2431, *SCI*3412, *SCI*3421

Table 5 shows the sensitivity of the actual and robust accuracies for the SCI fuser with respect to the orders of sensors

From Table 5, we see that all values of $\mathrm{tr}P_{SCIijkr}$ or $\mathrm{tr}{\overset{¯}{P}}_{SCIijkr}$ are close to these of $\mathrm{tr}P_{BCI}$ or $\mathrm{tr}{\overset{¯}{P}}_{BCI}$, respectively. This means that the robust or actual accuracies of the SCI fusers are not very sensitive to the orders of sensors.

## 8. Conclusions

Sequential covariance intersection fusion robust time-varying Kalman filters are presented for the multi-sensor systems with uncertainties of noise variances, the main contributions of this paper are as follows:

A minimax robust estimation approach of designing the robust local, BCI and SCI fused Kalman filters has been presented for the multisensor system with uncertain noise variances. For the multisensor time-invariant systems with uncertain noise variances, the convergence problem of the robust local and fused time-varying Kalman filters has been solved. The robust local, BCI and SCI fused steady-state Kalman filters have been presented by replacing the time-varying gains, variances and cross-covariances with their limits, respectively. The convergence in a realization of the local and fused time-varying and steady-state Kalman filters was proved by the dynamic error system analysis (DESA) method [39] and the dynamic variance error system analysis (DVESA) method [40].

The proposed results can be applied to some simulation application research, including target tracking systems, uninterruptible power supply systems, mass spring random vibration systems, and so on. The proposed results are limited to multisensor systems with uncertainties of noise variances. The extensions of the proposed results to multisensor systems with uncertainties of both model parameters and noise variances are under investigation.

## Figures and Tables

**Table 1:** The frequently used notations.

Name	Summary
t	the discrete time
Φ(t)	the state transition matrix
Γ(t)	the input transition matrix
$H_{i}\left(t\right)$	the measurement transition matrix.
∥A∥	the norm of matrix A.
k	the discrete time
E	the mathematical expectation operator
$A^{T}$	transpose of matrix A
$\delta_{ij}$	the Kronecker δ $\;\mathrm{function},\;\delta_{ii}=1,\delta_{ij}=0\left(i\neq j\right)$
trA	the trace of a matrix A
s	“steady-state”
“i.a.r”	the convergence in a realization

**Table 2:** The comparison of the computational loads.

robust BCI filter	$P_{BCI}\left(t|t\right)$	${\left((N+1)n\right)}^{3}$
robust SCI filter	$P_{SCI}\left(t|t\right)$	$n^{3}N$

**Table 3:** The accuracy comparison of local and fused robust time-varying Kalman filters at t=10.

$\mathrm{tr}P_{1}$	$\mathrm{tr}P_{2}$	$\mathrm{tr}P_{3}$	$\mathrm{tr}P_{BCI}$	$\mathrm{tr}P_{SCI123}$	$\mathrm{tr}P_{SCI132}$	$\mathrm{tr}P_{SCI231}$
0.6289	0.6972	0.4784	0.3839	0.4318	0.3888	0.3888
$\mathrm{tr}{\overset{¯}{P}}_{1}$	$\mathrm{tr}{\overset{¯}{P}}_{2}$	$\mathrm{tr}{\overset{¯}{P}}_{3}$	$\mathrm{tr}{\overset{¯}{P}}_{BCI}$	$\mathrm{tr}{\overset{¯}{P}}_{SCI123}$	$\mathrm{tr}{\overset{¯}{P}}_{SCI132}$	$\mathrm{tr}{\overset{¯}{P}}_{SCI231}$
0.5147	0.5719	0.4132	0.1813	0.1818	0.1905	0.1905

**Table 4:** The robust accuracy comparison of local and fused steady-state Kalman filters.

$\mathrm{tr}P_{1}$	$\mathrm{tr}P_{2}$	$\mathrm{tr}P_{3}$	$\mathrm{tr}P_{BCI}$	$\mathrm{tr}P_{SCI123}$	$\mathrm{tr}P_{SCI132}$	$\mathrm{tr}P_{SCI231}$
0.5538	0.5245	0.4390	0.3602	0.3971	0.3648	0.3648
$\mathrm{tr}{\overset{¯}{P}}_{1}$	$\mathrm{tr}{\overset{¯}{P}}_{2}$	$\mathrm{tr}{\overset{¯}{P}}_{3}$	$\mathrm{tr}P_{BCI}$	$\mathrm{tr}{\overset{¯}{P}}_{SCI123}$	$\mathrm{tr}{\overset{¯}{P}}_{SCI132}$	$\mathrm{tr}{\overset{¯}{P}}_{SCI231}$
0.4465	0.3815	0.3723	0.1717	0.1759	0.1795	0.1795

**Table 5:** The sensitivity of the actual and robust accuracies for the SCI fuser with respect to the orders of sensors.

$\mathrm{tr}P_{1}$	$\mathrm{tr}{\overset{¯}{P}}_{1}$	$\mathrm{tr}P_{2}$	$\mathrm{tr}{\overset{¯}{P}}_{2}$	$\mathrm{tr}P_{3}$	$\mathrm{tr}{\overset{¯}{P}}_{3}$	$\mathrm{tr}P_{4}$	$\mathrm{tr}{\overset{¯}{P}}_{4}$	$\mathrm{tr}P_{BCI}$	$\mathrm{tr}{\overset{¯}{P}}_{BCI}$
0.5538	0.4465	0.5245	0.3815	0.4390	0.3723	0.4786	0.4026	0.3312	0.1231
$\mathrm{tr}P_{SCI1234}$	$\mathrm{tr}{\overset{¯}{P}}_{SCI1234}$	$\mathrm{tr}P_{SCI1243}$	$\mathrm{tr}{\overset{¯}{P}}_{SCI1243}$	$\mathrm{tr}P_{SCI1324}$	$\mathrm{tr}{\overset{¯}{P}}_{SCI1324}$	$\mathrm{tr}P_{SCI1342}$	$\mathrm{tr}{\overset{¯}{P}}_{SCI1342}$	$\mathrm{tr}P_{SCI1423}$	$\mathrm{tr}{\overset{¯}{P}}_{SCI1423}$
0.3622	0.1207	0.3675	0.1407	0.3547	0.1325	0.3312	0.1611	0.3639	0.1482
$\mathrm{tr}P_{SCI1432}$	$\mathrm{tr}{\overset{¯}{P}}_{SCI1432}$	$\mathrm{tr}P_{SCI2314}$	$\mathrm{tr}{\overset{¯}{P}}_{SCI2314}$	$\mathrm{tr}P_{SCI2341}$	$\mathrm{tr}{\overset{¯}{P}}_{SCI2341}$	$\mathrm{tr}P_{SCI2413}$	$\mathrm{tr}{\overset{¯}{P}}_{SCI2413}$	$\mathrm{tr}P_{SCI2431}$	$\mathrm{tr}{\overset{¯}{P}}_{SCI2431}$
0.3639	0.1482	0.3547	0.1395	0.3547	0.1325	0.3639	0.1482	0.3312	0.1611
$\mathrm{tr}P_{SCI3412}$	$\mathrm{tr}{\overset{¯}{P}}_{SCI3412}$	$\mathrm{tr}P_{SCI3421}$	$\mathrm{tr}{\overset{¯}{P}}_{SCI3421}$						
0.3312	0.1611	0.3312	0.1611						
